# Improved Culture-Based Isolation of Differentiating Endothelial Progenitor Cells from Mouse Bone Marrow Mononuclear Cells

**DOI:** 10.1371/journal.pone.0028639

**Published:** 2011-12-28

**Authors:** Haruki Sekiguchi, Masaaki Ii, Kentaro Jujo, Ayumi Yokoyama, Nobuhisa Hagiwara, Takayuki Asahara

**Affiliations:** 1 Group of Vascular Regeneration Research, Institute of Biomedical Research and Innovation, RIKEN Center for Developmental Biology, Kobe, Japan; 2 Department of Cardiology, Tokyo Women's Medical University, Tokyo, Japan; 3 Yokohama Medical Center, National Hospital Organization, Kanagawa, Japan; 4 Group of Translational Stem Cell Research, Department of Pharmacology, Osaka Medical College, Osaka, Japan; 5 Regenerative Medicine Science, Tokai University, Kanagawa, Japan; Centro Cardiologico Monzino, Italy

## Abstract

Numerous endothelial progenitor cell (EPC)-related investigations have been performed in mouse experiments. However, defined characteristics of mouse cultured EPC have not been examined. We focused on fast versus slow adherent cell population in bone marrow mononuclear cells (BMMNCs) in culture and examined their characteristics. After 24 h-culture of BMMNCs, attached (AT) cells and floating (FL) cells were further cultured in endothelial differentiation medium separately. Immunological and molecular analyses exhibited more endothelial-like and less monocyte/macrophage-like characteristics in FL cells compared with AT cells. FL cells formed thick/stable tube and hypoxia or shear stress overload further enhanced these endothelial-like features with increased angiogenic cytokine/growth factor mRNA expressions. Finally, FL cells exhibited therapeutic potential in a mouse myocardial infarction model showing the specific local recruitment to ischemic border zone and tissue preservation. These findings suggest that slow adherent (FL) but not fast attached (AT) BMMNCs in culture are EPC-rich population in mouse.

## Introduction

Endothelial progenitor cells (EPCs) were first isolated from adult human peripheral blood in 1997[1]. Since the discovery, numerous EPC-related animal experiments for neovascularization in ischemic tissues have been performed as well as clinical studies with human EPCs for ischemic diseases. Among animal EPCs, mouse EPCs have been frequently used for understanding the cell biology and pathophysiological roles in ischemic tissues.

Originally, EPCs were recognized with dual profiles of immature cell, as stem or progenitor cell, and endothelial lineage cell, in terms of both marker expressions, i.e. Sca-1/Flk-1 [2], CD34/Flk-1 [3], c-kit/CD31 [4], c-kit/Tie-2 [5], CXCR4/Flk-1 [6], and Flk-1/E-cadherin [7], etc. has been used for mouse EPC identification/isolation from peripheral blood [8] or bone marrow (BM) mononuclear cells (MNCs) by fluorescence-activated cell sorter (FACS), indicating that cell surface marker-based definition of EPC is still controversial. On the other hand, cultured EPCs have also been used in a number of investigations because of its methodological simple and convenience for cell isolation. Conventionally, cultured EPCs can be isolated as an adherent cell population from cultured BMMNCs with endothelial differentiation medium, and the BM-derived adherent cells exhibiting endothelial characteristics such as acetylated low density lipoprotein (acLDL) uptake and lectin binding have been considered as cultured EPCs. [9] However, recent studies in which cultured EPCs are examined by FACS and molecular analysis for specific gene expressions have demonstrated that cultured EPCs is a heterogenous cell population mixed with or similar to other CD45+/CD11b+ hematopoietic lineage cells i.e. monocyte/macrophages. Thus, the development of another cell culture system that allows us to obtain more defined cultured EPCs is required for efficient therapeutic angiogenesis. One recent rat study [10] has clearly demonstrated that slow adherent cells could finally differentiate into a variety of endothelial marker expressing cells when freshly isolated BMMNCs were cultured in endothelial differentiation medium for 48 hours. These findings suggest that immature stem or progenitor cells exhibit its characteristics of slow adhesion activity in culture.

Based on the above evidences, we focused on fast adherent cells versus slow adherent cells following mouse BMMNC culture aiming to obtain defined mouse cultured EPCs without using complicated surface marker-dependent isolation method. In the present study, we separated freshly isolated mouse BMMNCs to fast adherent cells and slow adherent cells in culture, and characterize the cells by immunocytological and molecular biological analyses optimizing culture conditions. We also examined the cell functions for endothelial cells under a variety of physiological conditions. Finally, we explored not only pathophysiological roles but also therapeutic efficacy of the cells in sites of ischemia using a mouse myocardial infarction model.

## Results

### Differential cell characteristics and functions in FL cells versus AT cells

We separated BMMNCs into fast attach (AT) cell population and floating (FL) cell population 24 hours after seeding on matrix-coated culture plate with 10%FBS/DMEM, and further cultured for 3 days. Cell characteristics and functions were examined in attached BMMNCs (AT) and floating BMMNCs (FL) at 24 hour in culture, and total BMMNCs (TT) at 4 days in culture were used as a control.

First, we examined cell surface expressions in AT cells and FL cells under several medium conditions after initial 24-hour culture by FACS analysis (Table 1). In 10%FBS/DMEM with low glucose medium rather than the other medium, FL exhibited relatively high immature marker expressions whereas AT expressed several monocyte/macrophage markers. Interestingly, BMMNCs expressed more number of monocyte/macrophage markers in both AT cells and FL cells with RPMI 10%FBS+1×10^−5^M of 2ME+25ng/ml of GM-CSF medium, (Table S1) suggesting that BMMNCs committed to macrophage lineage in the condition of macrophage differentiation culture. We then examined cell surface makers in TT cells, AT cells and FL cells in 10%FBS/DMEM with low glucose medium by FACS analysis. (Figure S5) FL cells frequently expressed immature marker Sca-1 and c-kit at day1 and potential endothelial marker CD31 at day1 and day7 compared with AT cells. In contrast, AT cells frequently expressed macrophage maker CD11b and F4/80 compared with FL cells at day1 and day7. (Table1)

**Table 1:** Cell surface marker-based characterization of BMMNCs by FACS analysis.

Cell Type	Culture (days)	Sca-1	c-Kit	Flk-1	CD34	CD31	VE-Cad	CD45	CD11b	F4/80	CD14
**TT**	0	8.1±3.8	26.2±12.2	0.7±0.3	9.7±1.3	31.2±2.6	1.4±0.7	99.3±0.5	71.2±10.9	2.0±0.9	0.1±0.1
**TT**	1	0.4±0.3	0.8±0.3	0.2±0.1	0.2±0.1	3.6±2.4	7.4±3.3	93.8±4.3	87.1±7.1	7.5±5.5	3.6±2.4
**AT**	1	6.3±1.3	13.3±7.3	1.6±0.5	1.8±0.3	7.2±3.7	4.0±1.3	98.7±0.2	85.0±10.9	16.7±4.5	7.9±6.4
**AT**	7	0.5±0.1	1.3±0.3	0.2±0.1	0.4±0.3	1.7±1.2	3.1±1.3	87.1±7.1	79.7±15.4	17.4±15.4	5.5±4.3
**FL**	1	10.0±3.9*	21.2±9.1*	0.7±0.3	3.3±0.8*	15.4±5.5*	1.3±0.4	98.0±3.1	69.2±12.9*	4.0±1.8*	0.9±0.7*
**FL**	7	0.4±0.3NS	2.0±1.6**	0.2±0.1	0.8±0.6**	9.8±1.3**	3.7±1.0	92.1±7.9	51.6±13.2**	4.4±2.1**	0.7±0.5**

TT: freshly isolated total Bone marrow mononuclear cells (BMMNCs), AT: attached BMMNCs 24 hours after culture, FL: floating BMMNCs 24 hours after culture. Values are expressed as mean ± SEM (%).

*P<0.05 vs. AT (day 1); NS and

**P<0.05 vs. AT (day 7), n = 3.

**Figure 1:**
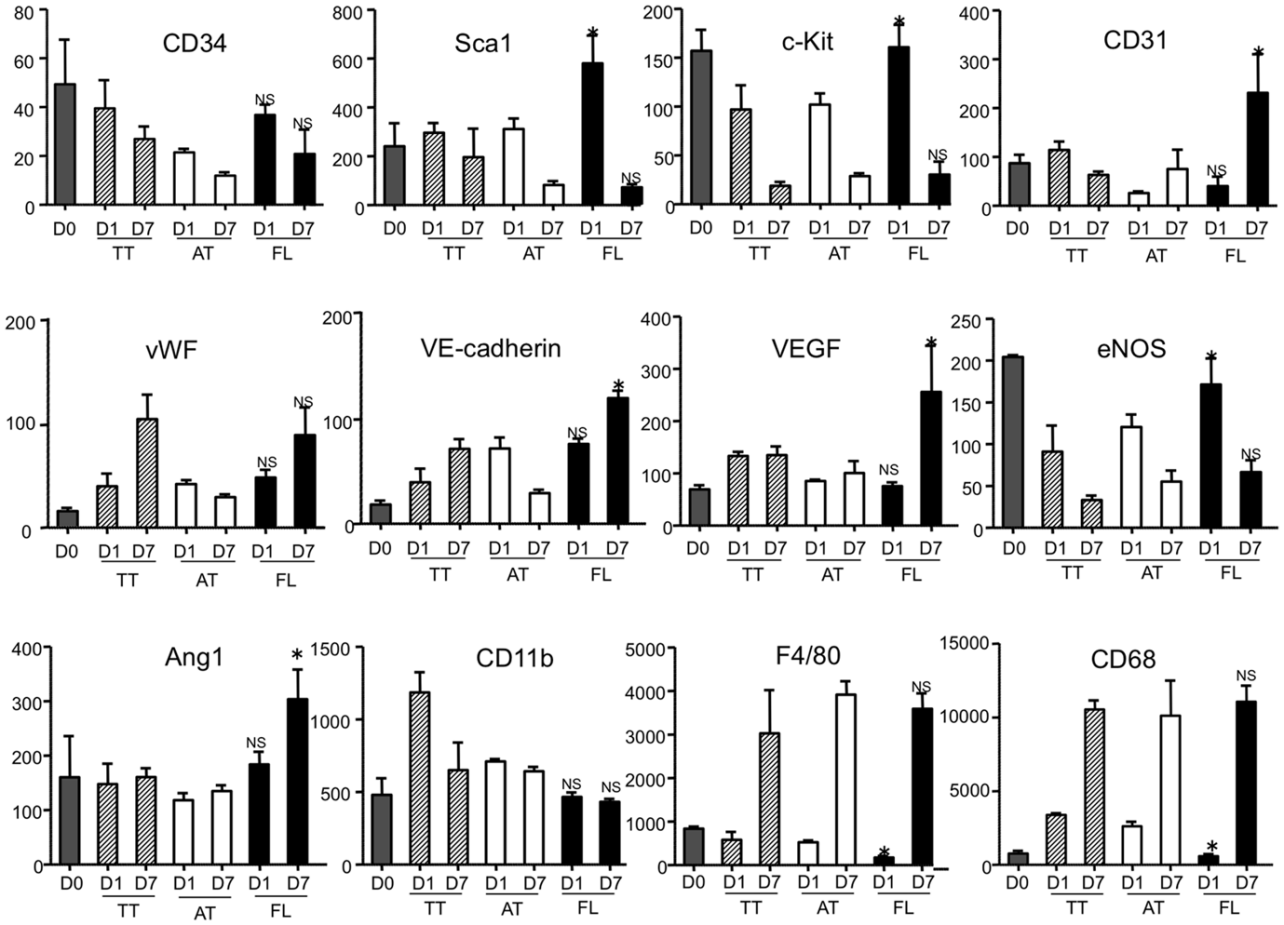
FL cells rather than AT cells exhibit EPC-like mRNA expression pattern following endothelial differentiation culture. BMMNCs are separated to attached (AT) cells and floating (FL) cells 24 hour (one day, D1) after culture with 10%FBS/DMEM medium on temperature-sensitive culture plate followed by further culture with 10%FBS/EBM2 growth factor containing medium for 6 days (D7). Real-time RT-PCR analysis was performed for the indicated genes at each time point. The freshly isolated total BMMNCs (D0) and non-separated total adherent cells (TT) were used as controls. Y-axis: relative mRNA expression normalized to GAPDG. Each gene expressions are indicated as relative mRNA expressions to GAPDH. NS and *, *P*<0.05: FL vs. AT at each time point.

We also examined mRNA expressions of TT cells, AT cells and FL cells. Consistent with the results of FACS analysis, immature marker Sca-1 and c-Kit expressions were significantly greater in FL cells than those in AT cells at day1. Moreover, endothelial lineage-related genes of CD31, VE-cadherin, VEGF, and Ang1 were highly expressed in FL cells compared with AT cells at day7. (CD31: AT, 75.3±52.8 vs. FL 231.1±113.4, VE-cadherin: AT, 29.9±1.9 vs. FL, 121.9±8.7, VEGF: AT, 100.5±39.0 vs. FL 289.3±121.8, and Ang1: AT, 101.7±58.1 vs. FL, 303.4±79.3, P<0.05 in each comparison) However, monocyte/macrophage-related genes of F4/80 and CD68 expressions exhibited no significant difference between in FL cells and in AT cells at day7, (F4/80: AT, 3985.0±102.8 vs. FL, 3735.6±236.6 and CD68: AT, 10093.6±3352.6 vs. FL 11067.0±1551.0, NS and NS, respectively) although the expressions were significantly reduced in FL cells compared with AT cells at day1. (F4/80: AT, 523.9±18.0 vs. FL, 171.3±38.8, CD68 and AT, 2601.5±364.2 vs. FL, 599.0±214.9, P<0.05 and P<0.05, respectively) (Figure 1).

### FL cells exhibit high potential of vasculogenic and angiogenic activities

**Figure 2:**
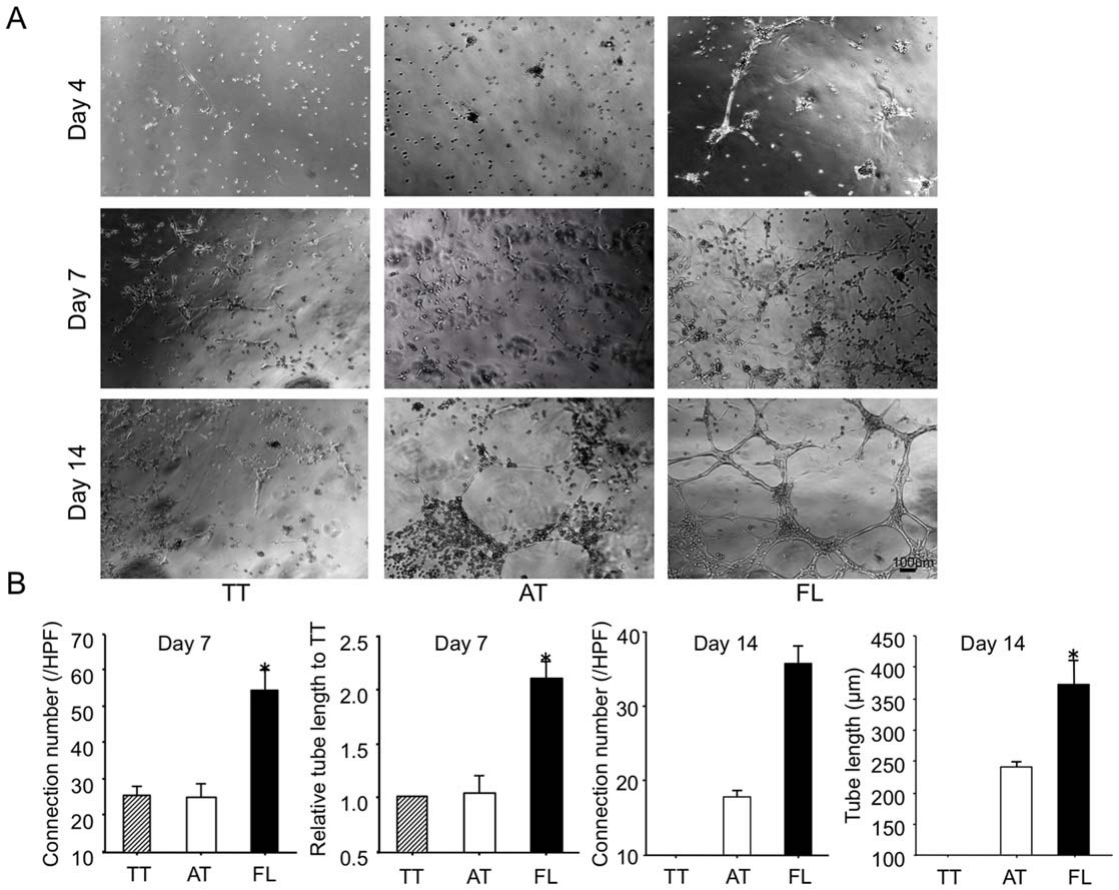
FL cells but not AT cells have a capacity to form massive tubes in vitro. BMMNCs are separated to attached (AT) cells and floating (FL) cells 24 hour after culture with 10%FBS/DMEM medium on temperature-sensitive culture plate followed by further culture with 10%FBS/EBM2 growth factor containing medium for 3 days. The non-separated total adhered cells (TT) were used as a control. The TT cells, AT cells and FL cells were further cultured with 10%FBS/EBM2 on Matrigel^TM^ coated 6-well plate. (A) Tube formation was evaluated under a phase-contrast microscope 4 (Day 4), 7 (Day 7), and 14days (Day 14) after culture on Matrigel^TM^. (B) Connection number in tubes and tube length 7 days (Day 7) and 14 days (Day 14) after culture are counted and measured, respectively, in each well and averaged (n = 3). *, *P*<0.05 vs. AT.

We performed tube formation assay with TT cells, AT cells and FL cells. TT cells transiently formed tube-like structure at day7 during 14 days in culture. AT cells failed to form tube-like structures until day 7 in culture although slight cell clusters could be observed at day 14. On the other hand, FL cells started to form tube-like structure and it was further expanded with massive tubes in the indicated time course up to day 14. (Figure 2A) Quantitative analysis for connection number and tube length in each group demonstrated that both parameters were significantly greater in FL cells than those in the other cells. (AT, 247.3±6.7 vs. FL, 360.3±46.8 µm, P<0.05) (Figure 2B).

**Figure 3:**
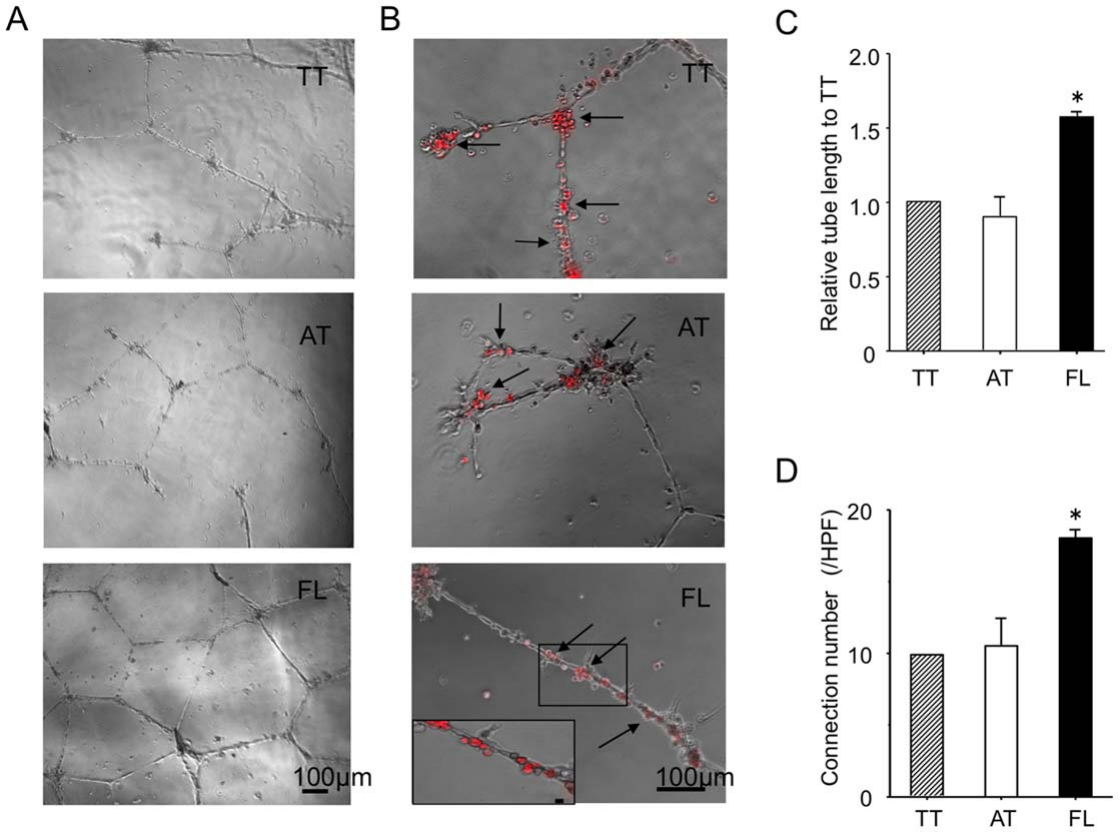
FL cells incorporated into HUVEC-derived tube-like structure as well as connections and enhanced tube formation. BMMNCs are separated to attached (AT) cells and floating (FL) cells 24 hour after culture with 10%FBS/DMEM medium on temperature-sensitive culture plate followed by further culture with 10%FBS/EBM2 growth factor containing medium for 3 days. The non-separated total adhered cells (TT) were uses as a control. HUVECs and DiI-acLDL-labeled TT cells, AT cells or FL cells are co-cultured with 10%FBS/EBM2 on Matrigel^TM^ coated 12-well plate. After 24 hours in culture, incorporation of each DiI-ac LDL-labeled cells was observed under a phase-contrast/fluorescent microscope. (A), Phase-contrast images. (B), Merged images of phase-contrast and fluorescent images. Arrows indicate DiI+ cells. One selected field is magnified in the left lower corner in the FL (B) image. Arrow indicates DiI+ cells. (C) Tube length and connection number were measured and counted, respectively, 48 hours after culture. Relative values of tube length to TT cells (C) and that of connection number to TT cells (D) were calculated and averaged. (n = 3) *, *P*<0.05 vs. AT. All experiments were performed in triplicate and confirmed the reproducibility.

We also examined each type of DiI-labeled cell incorporation into tube-like structure with mature endothelial cells (HUVECs) and its pro-angiogenic activity in vitro. AT cells were mainly incorporated into sites of connection, whereas FL cells mainly incorporated into trunks of tube. We observed incorporated cells both in sites of connection and in trunk of tube in TT cells. (Figure 3B, arrows) Quantitative analysis for connection number and tube length in each group also demonstrated that both parameters were significantly greater in FL cell group than those in the other cell group. (tube length: AT, 0.89±0.12 vs. FL 1.57±0.04 and Connection number: AT, 10.5±3.7 vs. FL 18±1.0, P<0.05 and P<0.05, respectively) (Figure 3A, C, and D).

### Shear stress influences FL cell phenotype as an endothelial lineage

**Figure 4:**
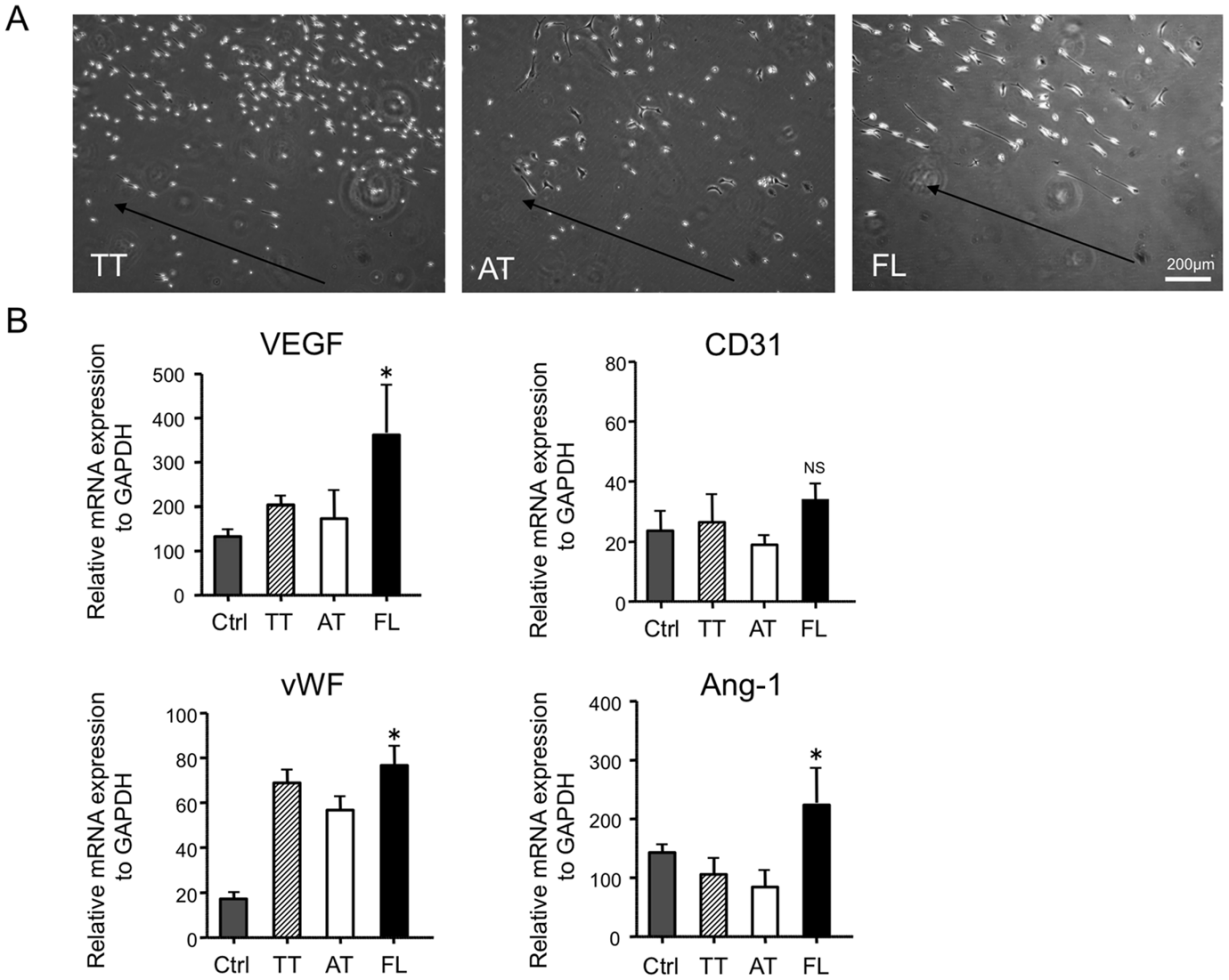
FL cells changed the morphology into spindle shape and expressed angiogenesis-related genes under shear stress. BMMNCs are separated to attached (AT) cells and floating (FL) cells 24 hour after culture with 10%FBS/DMEM medium on temperature-sensitive culture plate followed by further culture with 10%FBS/EBM2 growth factor containing medium for 3 days. The non-separated total adhered cells (TT) were uses as a control. (A) Cells were then cultured under circular flow for 48 hours. Morphological changes were observed under a phase-contrast microscope. Arrow indicates the direction of medium flow. (B) Angiogenesis-related cytokine mRNA expressions in morphologically changed cells under shear stress were analyzed by real-time RT-PCR and triplicated. Cells cultured without shear stress were used as a control (Ctrl). Relative mRNA expressions of each gene normalized to GAPDH are indicated in the graphs. *, *P*<0.05 vs. AT.

Since shear stress is known to enhance endothelial differentiation and stimulate cytokine release, we examined phenotypic alteration of each cell types under a shear stress by real-time RT-PCR analysis. After 48 hours in culture with shear stress, most of the FL cells changed its morphology to spindle shape along with the medium flow direction, however, only a few AT cells did and no dramatic change of the morphology was observed in most of the AT cells. As we expected, mixture of spindle shaped cells and non-spindle shaped cells could be observed in TT cells. (Figure 4A, arrows).

We also examined mRNA expressions of angiogenesis-related cytokines 48 hour-culture with shear stress in each group. Although there was no significant change in CD31 expression among all groups, FL cells expressed significantly high expressions of VEGF, vWF and Ang-1 compared with the other groups, (Figure 4B) indicating that FL cells exhibited endothelial-like characteristics.

### Less frequent EPC colony forming unit in FL and no colony forming unit in AT

An EPC-colony forming assay (CFA) was recently established in our laboratory. EPC-CFA using murine EPC enriched population, c-Kit+/Sca-1+/lineage negative (KSL) cells, identified two unique colonies (small EPC and large EPC colonies) that likely represent two distinct EPC populations, primitive (small) and definitive (large) EPC colony forming units, respectively. Small EPC colony forming cells, featured as “primitive EPCs”, are immature and proliferative, whereas large EPC colony forming cells or “definitive EPCs” are more prone to differentiation and contribute to vasculogenesis. [11], [12], [13], [14]

**Figure 5:**
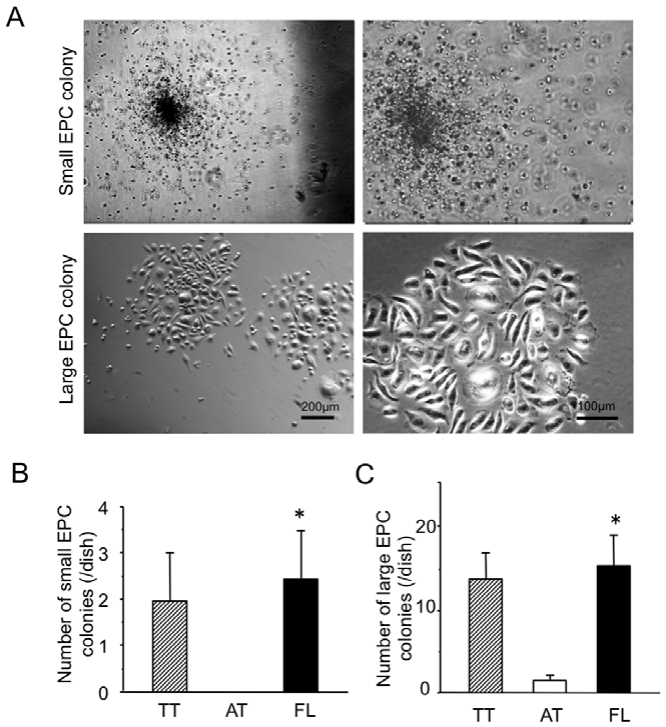
FL cells form Large EPC colonies rather than small EPC colonies. BMMNCs are separated to attached (AT) cells and floating (FL) cells 24 hour after culture with 10%FBS/DMEM medium on temperature-sensitive culture plate followed by further culture with 10%FBS/EBM2 growth factor containing medium for 3 days. The non-separated total adhered (TT) cells were uses as a control. Cells were cultured with methylcellulose-based culture medium in 6-well plate for 14 days for colony formation. (A) Two different colony types consisted of small size EPCs (small EPC colony) and large size EPCs (large EPC colony) can be observed under a phase-contrast microscope. Small EPC colony number (B) and large EPC colony number (C) were counted separately and averaged. (n = 3) *, *P*<0.05 vs. AT. All experiments were performed in triplicate and confirmed the reproducibility.

Interestingly, EPC-CFA represented a couple of primitive EPC colonies and definitive EPC colonies only from TT cells (1.7±1.5 and 0.3±0.6, respectively) or FL cells (1.6±0.6 and 1.6±0.6, respectively) ,but nothing form AT cells (0±0 and 0±0, respectively). (Figure 5 A, B, C) These results indicate AL population does not include any immature EPCs, while FL and TT remain some EPC colony forming capacity. However, the number of EPC-CFUs is considerably less compared to the frequency of CFUs in c-Kit+/Sca-1+/lineage negative murine BM cells, reported by recent previous publications [11], [12], [13], [14].

### FL cells contribute to neovascularization in ischemic myocardium exhibiting therapeutic potential following myocardial infarction

**Figure 6:**
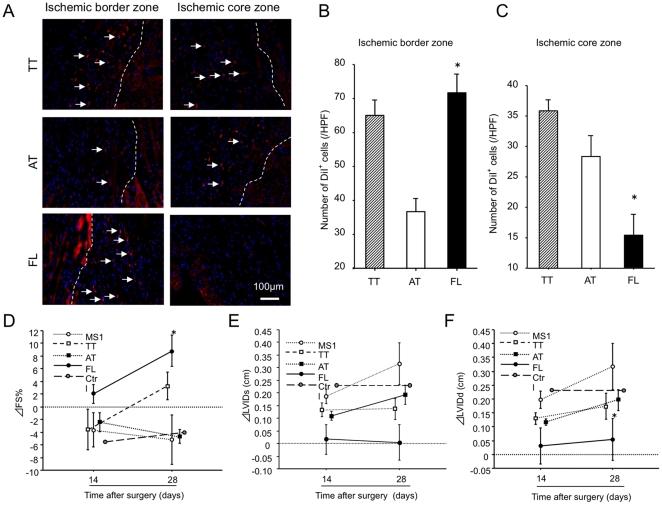
FL cells improve cardiac functional recovery with reduced myocardial infarction. DiI-acLDL labeled TT cells, AT cells or FL cells were injected to mice 3 days after induction of myocardial infarction, and the heart samples were harvested 3 days after cell injection. (A) The recruited DiI+ cells in ischemic border zone (left panels) and core zone (right panels) of myocardium were observed and counted under a fluorescent microscope. Dotted lines indicate the border of ischemic tissues and arrows indicate DiI+ cells. Each cell recruitment to ischemic myocardium was evaluated and expressed as a number of DiI+ cells in ischemic border zone (B) and in ischemic core zone (C) separately. *, *P*<0.05 vs. AT. Cardiac function was also evaluated by echocardiography 14 and 28 days after surgery. Increment in the parameter of fractional shortening (ΔFS) (D), left ventricular diameter at systole (ΔLVDs) (E), and left ventricular diameter at diastole (ΔLVDd) (F) were measured and averaged in the each cell-treated group. (n = 5) *, *P*<0.05 vs. AT. Mouse mature endothelial cells (MS-1) were used as another control.

Finally, to explore the pathophysiological behaviors of each cell types in ischemic tissues as endothelial progenitors we infused the cells systemically to mice following myocardial infarction (MI) and examined the cell recruitment and retention to ischemic myocardium histologically as well as cardiac functional recovery. Systemically injected DiI-acLDL labeled FL cells were frequently observed in ischemic border zone rather than ischemic core zone, while a large number of AT cells were in ischemic core zone rather than ischemic border zone. (Figure 6A) (FL cells vs. AT cells: ischemic border zone, 71.6±16.0 vs. 36.9±10.1/HPF, P<0.05 and ischemic core zone, 15.5±6.6 vs. 28.3±6.8/HPF, P<0.05). Although TT cells recruited to ischemic border zone at similar level to FL cells, the number of recruited TT cells was significantly higher than that of FL cells in ischemic core zone. (TT cells vs. FL cells: ischemic border zone, 65.1±13.7 vs. 71.6±16.0/HPF, NS and ischemic core zone, 35.8±3.9 vs. 15.5±6.6/HPF, P<0.05). (Figure 6B and 6C) These results clearly indicate that FL cells dominantly recruit to sites of ischemia where generally angiogenesis occurs and AT cells dominantly recruit to sites of necrosis where generally severe inflammation occurs, suggesting that FL cells and AT cells could more likely behave as EPCs and monocyte/macrophages in sites of ischemia, respectively.

Next, we evaluated the therapeutic effect of each type of cells on cardiac functional recovery following myocardial infraction 28 days after surgery by histological assessment and echocardiography. PBS (Ctrl) and MS-1 cell line, a mature endothelial cell line, were used as controls in this experiment. FL cells exhibited the best cardiac functional recovery with the following echocardiographical parameters, Δfractional shortening, (ΔFS: FL, 8.84±2.43 vs. TT, 3.27±2.15, AT −4.70±1.07, or MS-1 −5.22±3.87%, P<0.05 in each comparison) (Figure 6D) left ventricular internal diameter in systole, (ΔLVDIs: FL, 0.003±0.03 vs. TT, 0.14±0.04, AT, 0.20±0.06, or MS-1, 0.31±0.16cm, P<0.05) (Figure 6E) and left ventricular internal diameter in diastole, (ΔLVDd: FL, 0.04±0.07 vs. TT, 0.15±0.06, AT, 0.18±0.03, or MS-1, 0.30±0.16cm, P<0.05) (Figure 6F) compared with the other cell types.

**Figure 7:**
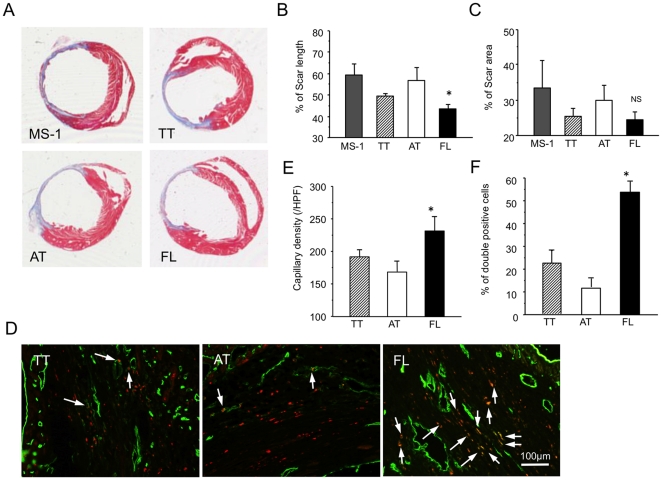
FL cells recruit to ischemic border zone rather than ischemic core zone promoting angiogenesis. (A) Heart sections were examined by Masson's Trichrome staining 28 days after surgery in the each cell-treated group (n = 5). Percent of scar (blue area) length in internal LV circumference (B) and percent of scar (blue) area in entire LV cross-sectional area (C) were calculated and averaged. (n = 5) NS and *, *P*<0.05 vs. AT. MS-1 cells were used as another control. (D) FITC-BS1 lectin perfused ischemic myocardium with recruited DiI+ cells was examined under a fluorescent microscope 7 days after cell injection (3 days following MI). Arrows indicate double positive staining area by FITC conjugated BS1-lectin (green) and DiI+ (red) positive cells. Capillaries (green dots) (E) and the percent of green and red double positive cells (yellow dots) in capillaries (F) were calculated in bilateral side of ischemic border zone in each sample, and the averaged values were further averaged for statistical analysis. (n = 5) *, *P*<0.05 vs. AT.

In histological analysis, we evaluated the scar size on heart sections with Masson's trichrome staining (Figure 7A). Consistent with the results in echocardiographical analysis, although there was no significant difference in % LV fibrosis area in entire LV cross-sectional area among all types of cell-treated mice, (FL: 24.5±3.9 vs. TT: 25.6±4.2, AT: 30.0±8.2, or MS-1: 33.6±14.8%, NS) (Figure 7C) the FL cells-treated mice exhibited significantly reduced both % of LV fibrosis length in entire LV circumference (FL: 43.3±3.2 vs. TT: 49.2±3.5, AT: 56.6±11.7, or MS-1: 59.2±10.0%, P<0.05) (Figure 7B) compared with the other cell type-treated mice. Also, we examined FITC (green) conjugated BS-1 lectin-perfused ischemic myocardium with DiI (red)-labeled cell injection under a fluorescent microscope. The BS-1 lectin/DiI double positive cells were frequently observed in the FL cells-treated mice than in the TT cells-treated mice and the AT cells-treated mice. (Figure 7D) The capillary density and the percent of incorporated DiI-labeled cells into capillaries in ischemic border zone of the FL cells-treated mice was significantly higher than those of the TT cells-treated and the AT cells-treated mice. (Capillary density: FL, 231.8±49.3 vs. TT, 190.4±30.6 or AT, 168.3±42.5/HPF, P<0.05 and Double positive cell #: FL, 53.8±7.8 vs. TT, 21.9±9.9 or AT, 11.2±6.4%, P<0.05) (Figure 7E and 7F).

These results indicate that FL cells rather than TT cells or AT cells differentiated into endothelial lineage in sites of angiogenesis and therefore might involve more endothelial progenitors which give rise to vasculogenesis and contribute to neovascularization in ischemic tissues resulting in preservation of damaged myocardium from ischemic insult and better cardiac functional recovery following MI.

## Discussion

In the present study, we have shown that slow adherent mouse BMMNCs in culture, FL cells, exhibited genetic phenotype of both immature and endothelial lineage cells, high potential of forming tube-like structure and spindle shape-morphologic change under a shear stress condition with increased angiogenic cytokine mRNA expressions in vitro, though less EPC colony forming activity was observed. The majority of FL cells have been shown to recruit to ischemic border zone and have a therapeutic effect on infarct heart limiting MI size and increasing angiogenesis/vasculogenesis with high cell incorporation into neovasculature as endothelial cells in ischemic myocardium. On the other hand, fast adherent cells in culture, AT cells, exhibited less genetic phenotype of either immature or endothelial lineage, low tube-like structure forming activity and no EPC colony forming activity, and no morphological change under a shear stress condition in vitro. Differing from FL cells, majority of AT cells recruited to ischemic core zone but not border zone and demonstrated little therapeutic effect on MI with less angiogenesis in ischemic myocardium. Overall, the mixed cell population of FL cells and AT cells, TT cells, also exhibited similar phenotypes and behavior in vitro and in vivo, respectively, to a lesser extent of FL cells. Taken together, FL cells appear to be compatible to EPC enriched BMMNC population and AT cells are considered to be monocyte/macrophage or inflammation-related cell-rich BMMNC population, while TT cells appear to be just mixed cell population of FL cells and AT cells.

Since the isolation of circulating EPCs in human PB,[1] the original culture-based method to obtain adherent human EPCs from CD34+ cells have been adapted to mouse EPC isolation from BMMNCs or PBMNCs for numerous vascular regeneration-related investigations. However, the characteristics/phenotype of mouse EPCs isolated by original culture-based method has not always been equivalent to endothelial progenitors but i.e. monocyte/macrophage-like cells,[15]and we first realized serum concentration-dependent EPC differentiation in mouse BMMNCs. (Figure S2A) Four days culture of BMMNCs in higher FBS concentrations gave rise to significantly increased number of EPCs which are defined by acLDL-uptake and BS-1 lectin binding. (2%FBS: 86±2% vs. 10%FBS: 97 ±1% and 20%FBS: 97 ±2%, P<0.05 and 2%FBS86±2% vs. 5%FBS: 91 ±3%, NS) (Figure S2B) Real-time RT-PCR analysis also showed that mouse BMMNCs cultured in 10%FBS medium up-regulated significantly higher mRNA expressions of SDF-1α, Ang-1, Ang-2, eNOS and IGF-1 than those in other FBS conditions, (Figure S3 and Table S1) suggesting that the BMMNCs cultured in 10%FBS medium exhibited the highest angiogenic potential among all FBS concentrations. We then decided to use 10%FBS culture condition throughout the present experiments.

An EPC-CFA was designed by our laboratory to elucidate EPC colony forming ability to detect immature EPC population [11], [12], [13], [14]. EPC-CFA using murine EPC enriched population, c-Kit+/Sca-1+/lineage negative (KSL) cells, identified two distinct EPC populations, primitive (small) and definitive (large) EPC colony forming units, respectively. Small EPC colony forming cells, featured as “primitive EPCs”, are immature and proliferative, whereas large EPC colony forming cells or “definitive EPCs” are more prone to differentiation and contribute to vasculogenesis. [11], [12], [13], [14] We also confirmed the phenotypic difference of small EPC colony and large EPC colony by real-time RT-PCR (Figure S1). EPC-CFA of TT, FL, and AT represented a small number of primitive and definitive EPC colony formation from TT cells or FL cells, while no colony formation was observed form AL cells. The number of EPC-CFUs from FL and TT (2∼3/5000cells) is considerably less compared to the frequency of CFUs in immature EPC-enriched population, such as KSL cells murine BM cells (20∼30/500 cells), reported by recent previous publications. [11], [12], [13], [14] This indicate that AT population does not include any immature EPC to give rise a colony, but FL and TT cell remain few capacity to form colonies though the main population are further differentiating not to form an EPC colony. Therefore, FL cells are likely enriched for differentiating EPCs that have a capacity for forming small/large EPC colonies with both stem/progenitor and endothelial mRNA expressions. (Figure S4) Also, TT cell characteristics appear to be combination with FL cell and AT cell characteristics. Indeed, our results of tube formation assay and recruitment to ischemic myocardium (Figure 3 and 6A, B, C) support the speculation.

In case of human EPCs, Chang et al. have recently classified PB-derived EPCs into early endothelial progenitor cells (Early EPCs) and late outgrowth endothelial cells (OECs) in culture system, demonstrating that early EPCs were more heterogeneous populations involving CD14+ cytokine-producing cells and CD14-/CD34+/KDR+ cells than OECs which are also derived from CD14- early EPCs expressing KDR. Although it would be difficult to simply compare the above human PB-derived EPC classification with our mouse BM-derived EPC (FL cell-derived), we have summarized the difference of culture-based human/mouse EPC characteristics in Table S2. Since FL cells showed superior endothelial functions releasing high levels of cytokine than AT cells and TT cells, mouse cultured FL cell-derived EPCs appear to exhibit both characteristics of human CD14- early EPCs and mature endothelial cells as human OECs[16]. In terms of other human cultured EPC classification, non- or weakly adherent round-shaped cell subpopulation of CD3+CD31+CXCR4+ angiogenic T lymphocytes in PB has been shown to be EPC-like characteristics.[17] In contrast, our FACS data showed that the percentages of CD3+ cells were 9.2% and 0.6% at day 4 and day 7 in cultured EPCs (TT cell-derived), respectively, suggesting that either FL cells or AT cells also involve few number of angiogenic CD3+ T lymphocytes. (Figure S6) Finally, we assume that this so-called adhesion-dependent mouse cultured EPC isolation method can also be adapted to human case as well, however, since the timing for AT cell exclusion in this method would be different among cell species i.e. initial 48-hour plating time is used for rat cultured EPC isolation,[10], [18] the separation timing of AT cells and FL cells in total MNCs should be optimized independently.

In summary, the modified culture-based mouse BM-derived EPC isolation method presented in this study clearly indicate that FL cells from BMMNCs are EPC enriched population and AT cells from BMMNCs are more likely monocyte/macrophage enriched population, although monocyte/macrophage markers such as CD11b, F4/80, and CD14 are expressed in some of the FL cells. (Table 1) The possible reasons why there are certain overlapped endothelial or monocyte/macrophage markers in each cell type might be because of: 1) the difficulty of complete separation between slow adherent cells and fast adherent cells and 2) the inclusion of some endothelial or monocyte/macrophage lineage uncommitted cells both in FL cells and in AT cells. Indeed, there are several reports in which mouse CD11b+ MNCs [19], [20] or human CD14+ MNCs [21], [22], [23] has been shown to differentiate into endothelial lineage exhibiting EPC-like characteristics.

In conclusion, we have newly developed culture-based mouse BM-derived EPC isolation method modifying an original one characterizing differentiating EPCs using a variety of assessments. This so-called “newly modified cultured EPC” methodology would clarify mouse EPC biology in physiological and pathological settings, and contribute to vascular regeneration researches.

## Materials and Methods

### Experimental animal model

Experiments were performed in 8-12 weeks old male WT (C57BL/6J) mice.

All surgical procedures were approved by the Institutional Animal Care and Use Committee of RIKEN center for developmental biology (approval ID:AH21-01) and were consistent with the *Guide for the Care and Use of Laboratory Animals*. Acute myocardial infarction was induced by permanent left anterior descending coronary artery ligation as described previously. [24] Briefly, mice were anesthetized with sodium pentobarbital (50 mg/kg IP) and orally intubated with a 23G IV catheter and artificially ventilated with a respirator (Harvard Apparatus, Holliston, MA). A left intercostal thoracotomy was performed and the ribs were retracted with 5-0 polypropylene sutures to open the chest. After the pericardium was opened, the left anterior descending (LAD) branch of the left coronary artery was ligated distal to the bifurcation between LAD and diagonal branch using 7-0 polypropylene sutures through a dissecting microscope. After positive end-expiratory pressure was applied to fully inflate the lung, the chest was closed with 6-0 polypropylene sutures.

Cells were injected to mice 3 days after MI induction via a tail vein for the assessment of either acute cell recruitment 24 hours after cell injection or cell incorporation into neovasculature 14 days after cell injection.

### Isolation of BMMNCs and separation of fast/slow adherent cells

Bone marrow mononuclear cells (BMMNCs) were isolated from mouse bones as described previously. [24] After isolation of BMMNCs, a part of the cells were examined by FACS analysis as freshly isolated BMMNCs. The left part of the cells was divided into 2 groups and seeded on: 1) rat vitronectin coated UpCell culture plate (CellSeed, Inc. Tokyo, Japan) with 10%FBS/EBM2 medium supplemented with growth factors (SingleQuots, Lonza) and cultured for 7days as total BMMNCs (TT cells) and 2) on Pronectin (Sigma) coated UpCell culture plate with 10%FBS/DMEM medium and separated attached and floating BMMNCs on new UpCell culture plate 24 hours after seeding. Both of the cells were further cultured in 10%FBS/EBM2 medium supplemented with growth factors (SingleQuots, Lonza) for 6 days. All floating cells at day 4 after initial cell plating were washed out with fresh culture medium, and fast (within 24 hours) adherent cells (AT cells) and slow (within 4 days) adherent cells (FL cells) were used for in vitro studies.

### Fluorescence-Activated Cell Sorting Analysis

To evaluate the surface marker expression of BMMNCs, the viable cell population was analyzed by FACS Aria (BD Biosciences) with the following antibodies: PE-conjugated anti-Sca-1 antibody, (mouse IgG2b, BD Pharmingen), PE-conjugated anti-c-Kit antibody (mouse IgG2b, Bio Legend), PE-conjugated anti-Flk-1 antibody, (mouse IgG2a, BD Pharmingen), FITC-conjugated anti-CD34 antibody (mouse IgG2a, BD Pharmingen), PE-conjugated anti-CD31 antibody (mouse IgG2a, BD Pharmingen), FITC-conjugated anti-VE-cadherin antibody (rabbit IgG, Alexis), PE-conjugated anti-CD45 antibody (mouse IgG2b, BD Pharmingen), FITC-conjugated CD11b antibody (mouse IgG2b, BD Pharmingen), FITC-conjugated anti-F4/80 antibody (mouse IgG2a, Abcam), PE-conjugated anti-CD14 antibody (mouse IgG1, BD Pharmingen), and PE-conjugated anti-CD3 antibody (mouse IgG2b, BD Pharmingen). Isotype-identical antibodies served as negative controls (Jackson ImmunoResearch).

### Shear stress test

TT cells, AT cells and FL cells cultured in 10%FBS/EBM2 medium supplemented with growth factors (SingleQuots, Lonza) on 35mm-culture dish were put on mini-magnet rotors (KENIS, Osaka, Japan) individually. Three sterilized small stirrers are put in each dish and rotated at 1500 rpm. After 48 hours in culture, cell morphology was observed under a phase contrast microscope and RNA samples were prepared for real-time RT-PCR analysis.

### EPC colony formation assay

TT cells, AT cells and FL cells (5000 cells/35mm-culture dish) were cultured in methyl cellulose based medium (MethoCult M3236, Stem Cell Technologies, Inc.) supplemented with VEGF (50ng/mL), SCF (100ng/mL), IL-3 (20ng/mL), EGF (50ng/mL), bFGF (50ng/mL), IGF-1 (50ng/mL) and 30% FBS for 7 days. Formed colonies were manually counted under a phase contrast microscope in entire filed of 35mm-culture dish.

### Real-time RT-PCR analysis

Total RNA was isolated from cell samples using RNAeasy Mini kit (QIAGEN) according to the manufacturer's instructions. cDNA was synthesized using PrimeScript RT reagent Kit: (TAKARA BIO Inc., Japan) primers and probe sequences are provided in Table S1. For quantitative RT-PCR, the converted cDNA samples (2 µl) were amplified in triplicate in a final volume of 10 µl using SYBR Green Master Mix reagent (Applied Biosystems) and gene-specific primers with an ABI Prism 7700 RT-PCR machine (Applied Biosystems). Melting curve analysis was performed with Dissociation Curves software (Applied Biosystems) and the mean cycle threshold [25_ENREF_25] values were used to calculate gene expression levels with normalization to mouse GAPDH.

### Assessment of vascularity in ischemic area

Functional vessels with blood flow were visualized by infusion of FITC-conjugated BS-1 lectin (Vector Laboratories) for 15 minutes before sacrifice, and capillary density was evaluated by counting green fluorescent tubular structures in bilateral ischemic border zone on one section and averaged. Five sections from 5 different mice were evaluated for capillary density.

### Statistical analysis

All values are expressed as mean ± SEM. Statistical analyses were performed with commercially available software (StatViewTM, Abacus Concepts, Berkeley, CA, USA); comparisons between two groups were tested for significance with a Mann-Whitney U-test, and comparisons between multiple groups were tested for significance via analysis of variance (ANOVA) followed by post-hoc testing with the Tukey procedure. A *P* value of less than 0.05 was considered significant.

## Supporting Information

Figure S1 **Small EPC colony expressed immature markers and the large EPC colony expressed differentiated endothelial markers.** Significant high mRNA expressions of Sca-1, CD34, c-Kit and Flk-1 were observed in small EPC colony and those of VE-cadherin, eNOS, bFGF and Ang-1 were observed in large EPC colony. BMMNCs are seeded in the methylcellulose-based medium and cultured for 14 days. Small EPC colonies (Small) and large EPC colonies (Large) are manually aspirated by glass capillary for real-time RT-PCR analysis. All mRNA expressions were normalized to GAPDH and presented in the graphs. * and NS, *P*<0.05 vs. Small. 2%FBS. ND: not detectable.
(TIFF)

Figure S2 **10%FBS is the best serum concentration for mouse EPC culture.** A**.** Mouse BMMNCs were seeded with EBM2 medium supplemented with growth factors and 2, 5, 10 or 20% FBS on Pronectin F coated culture dish. After 7 days in culture, the cells are stained with FITC-isolectin B4 and DiI-acLDL for EPC detection. B. The number of double positive cells was counted in each plate. (n = 3).
(TIFF)

Figure S3 **mRNA expressions of endothelial genes are up-regulated by hypoxic condition in EPCs cultured in 10%FBS medium.** The mRNA expressions of SDF-1α, Ang-1, Ang-2, eNOS and IGF-1 in BMMNCs were up-regulated by hypoxia in10%FBS/EBM2 culture medium. TT cells, AT cells and FL cells cultured in 2, 5,10 and 20%FBS/EBM2 medium for 4 days and further cultured under normoxic (20% O_2_) or hypoxic (5% O_2_) condition with new culture. After 48 hours in culture, the cells were harvested for real-time RT-PCR analysis. All mRNA expressions were normalized to GAPDH and presented as relative values in hypoxia to that in normoxia. *, *P*<0.05 vs. 2%FBS.
(TIFF)

Figure S4 **FL cell-derived EPC colonies exhibit EPC-like characteristics.** FL cell-derived small EPC colony expressed higher mRNA of stem/progenitors and FL cell-derived large EPC colony expressed higher mRNA of endothelial cell and cytokines than AT cell-derived colonies. TT cells, AT cells and FL cells are seeded in the methylcellulose-based medium and cultured. After 14 days in culture, small EPC colonies (Small) and large EPC colonies (Large) were manually aspirated by glass capillary for real-time RT-PCR analysis. All mRNA expressions were normalized to GAPDH and presented in the graphs. Y-axis: relative mRNA expression to GAPDH. * and NS, *P*<0.05 vs. AT.
(TIFF)

Figure S5 **Forward Scatter (FSC) and Side Scatter (SSC) in TT, AT and FL for FACS analysis.** After bone marrow derived mononuclear cells culture, the cells were harvested and examined the cell size by FSC and architecture by SSC to determine a certain cell population that is supposed to be analyzed for cell surface markers by FACS system. The gated cell population (green dots in R1 area) in each representative FSC/SCC graph was used for FACS analysis.
(TIFF)

Figure S6 **CD3 expression in TT cells at day4 and day7 after culture.** After 4 days and 7 days in culture, adherent bone marrow derived mononuclear cells (TT cells) were harvested and analyzed for CD3 expression by FACS analysis. The percentage of CD3 positive cells in day 4-TT cells (A) and day 7-TT cells (B) was indicated in each graph. Purple area, Isotype control PE conjugated IgG and Green line, PE conjugated anti-CD3 antibody.
(TIFF)

Table S1 **BMMNC surface marker expressions under different culture conditions.**
(TIFF)

Table S2 **Phenotypic comparison in mouse cultured FL-EPC vs. human cultured EPC.**
(TIFF)
