# Supplementary figures and images for: Improved Culture-Based Isolation of Differentiating Endothelial Progenitor Cells from Mouse Bone Marrow Mononuclear Cells
Source: PLoS One. 2011 Dec 28;6(12):e28639. doi: 10.1371/journal.pone.0028639 (PMC3247221; doi:10.1371/journal.pone.0028639)

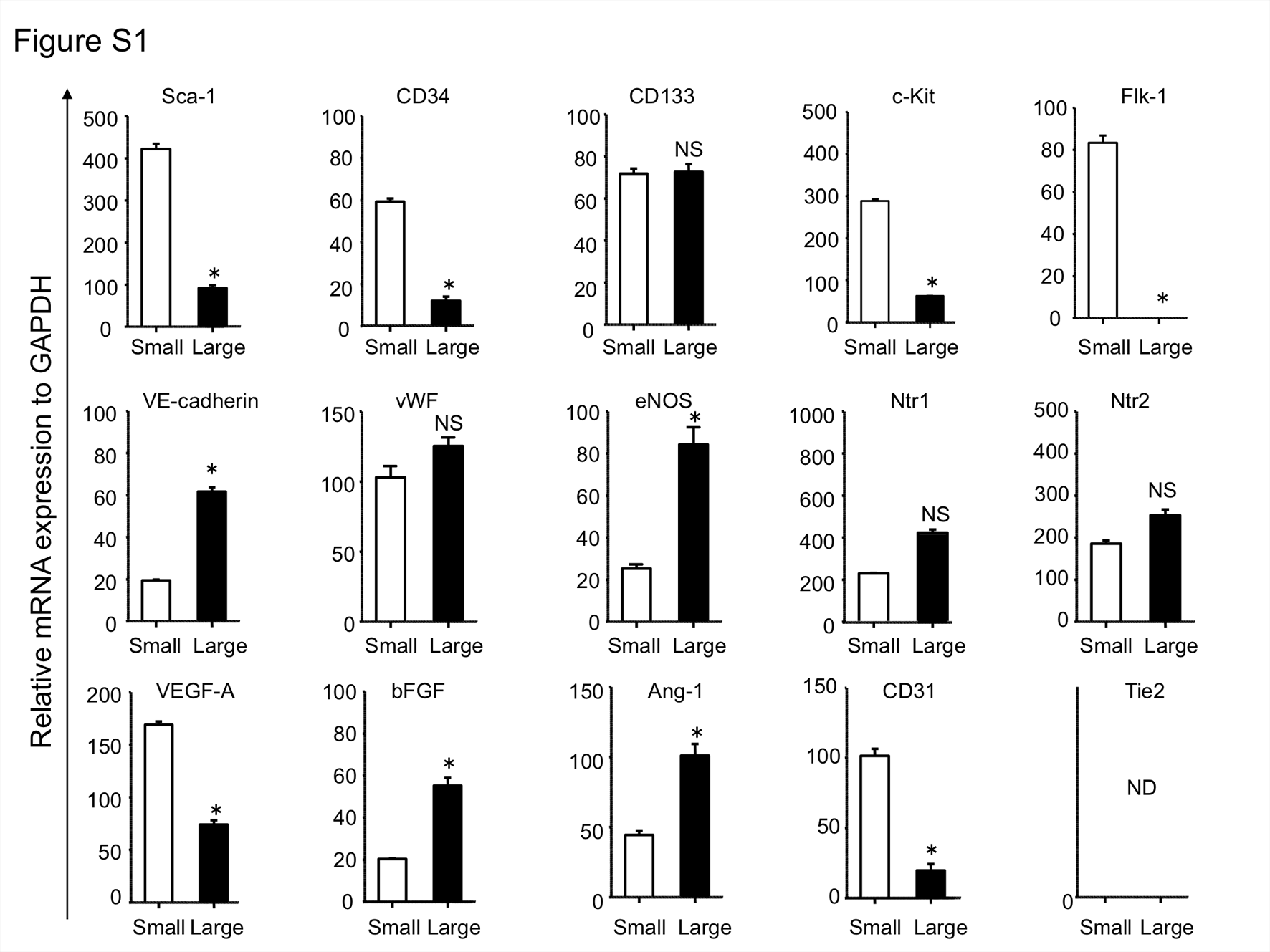

Supplement: Figure S1 — Small EPC colony expressed immature markers and the large EPC colony expressed differentiated endothelial markers. Significant high mRNA expressions of Sca-1, CD34, c-Kit and Flk-1 were observed in small EPC colony and those of VE-cadherin, eNOS, bFGF and Ang-1 were observed in large EPC colony. BMMNCs are seeded in the methylcellulose-based medium and cultured for 14 days. Small EPC colonies (Small) and large EPC colonies (Large) are manually aspirated by glass capillary for real-time RT-PCR analysis. All mRNA expressions were normalized to GAPDH and presented in the graphs. * and NS, P<0.05 vs. Small. 2%FBS. ND: not detectable. (TIFF) [file pone.0028639.s001.tif]

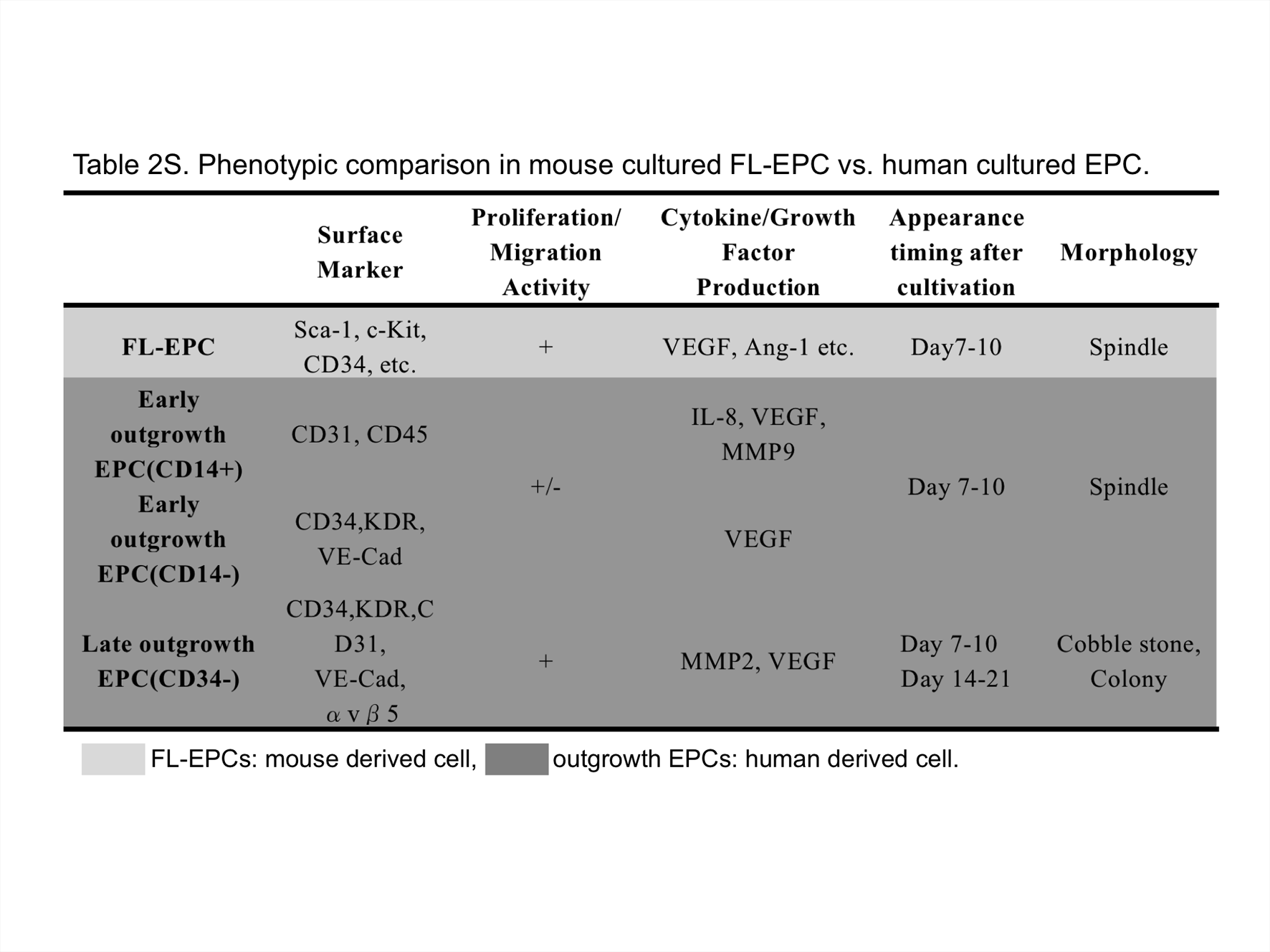

Supplement: Figure S2 — 10%FBS is the best serum concentration for mouse EPC culture. A. Mouse BMMNCs were seeded with EBM2 medium supplemented with growth factors and 2, 5, 10 or 20% FBS on Pronectin F coated culture dish. After 7 days in culture, the cells are stained with FITC-isolectin B4 and DiI-acLDL for EPC detection. B. The number of double positive cells was counted in each plate. (n = 3). (TIFF) [file pone.0028639.s002.tif]

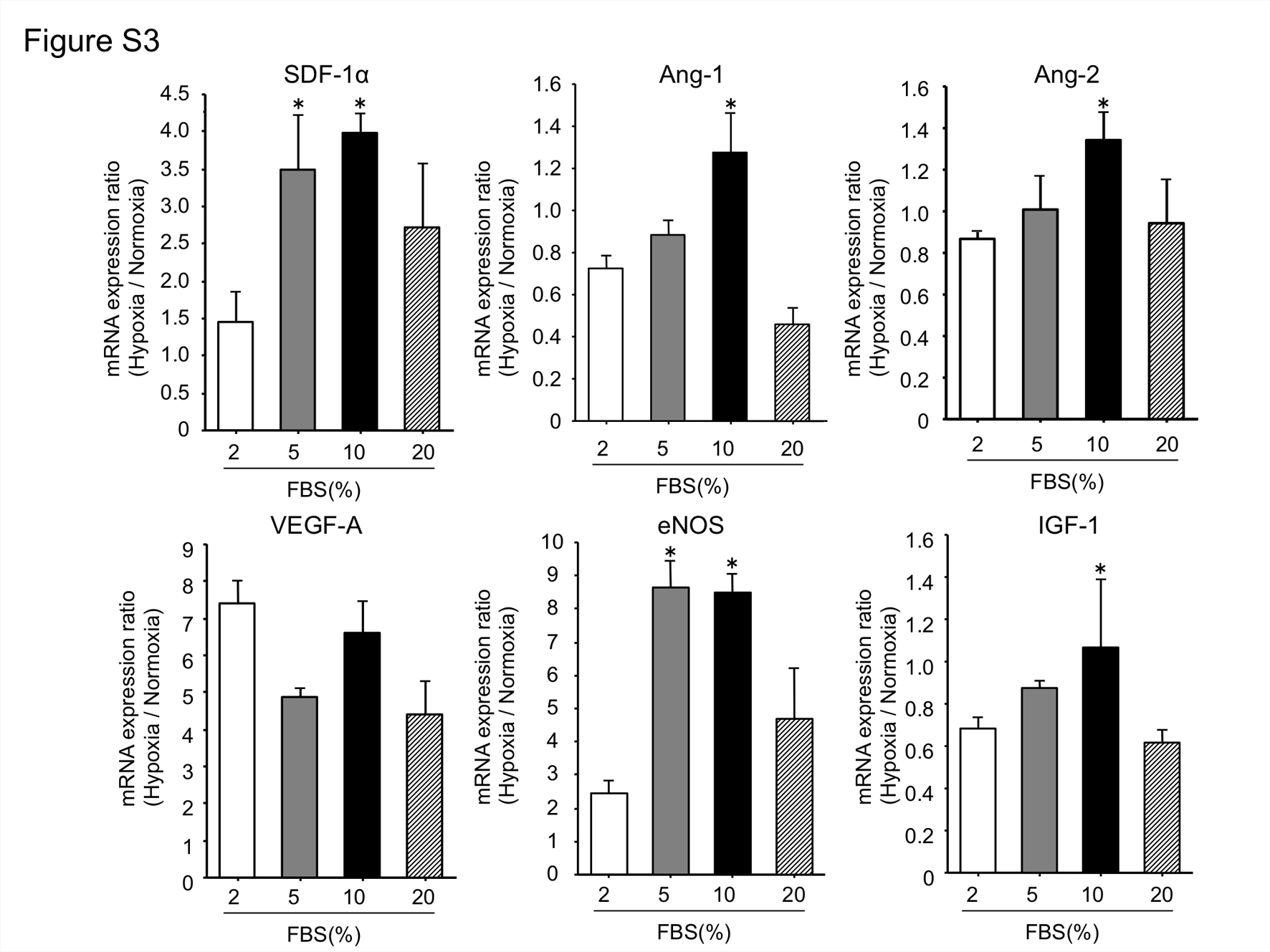

Supplement: Figure S3 — mRNA expressions of endothelial genes are up-regulated by hypoxic condition in EPCs cultured in 10%FBS medium. The mRNA expressions of SDF-1α, Ang-1, Ang-2, eNOS and IGF-1 in BMMNCs were up-regulated by hypoxia in10%FBS/EBM2 culture medium. TT cells, AT cells and FL cells cultured in 2, 5,10 and 20%FBS/EBM2 medium for 4 days and further cultured under normoxic (20% O2) or hypoxic (5% O2) condition with new culture. After 48 hours in culture, the cells were harvested for real-time RT-PCR analysis. All mRNA expressions were normalized to GAPDH and presented as relative values in hypoxia to that in normoxia. *, P<0.05 vs. 2%FBS. (TIFF) [file pone.0028639.s003.tif]

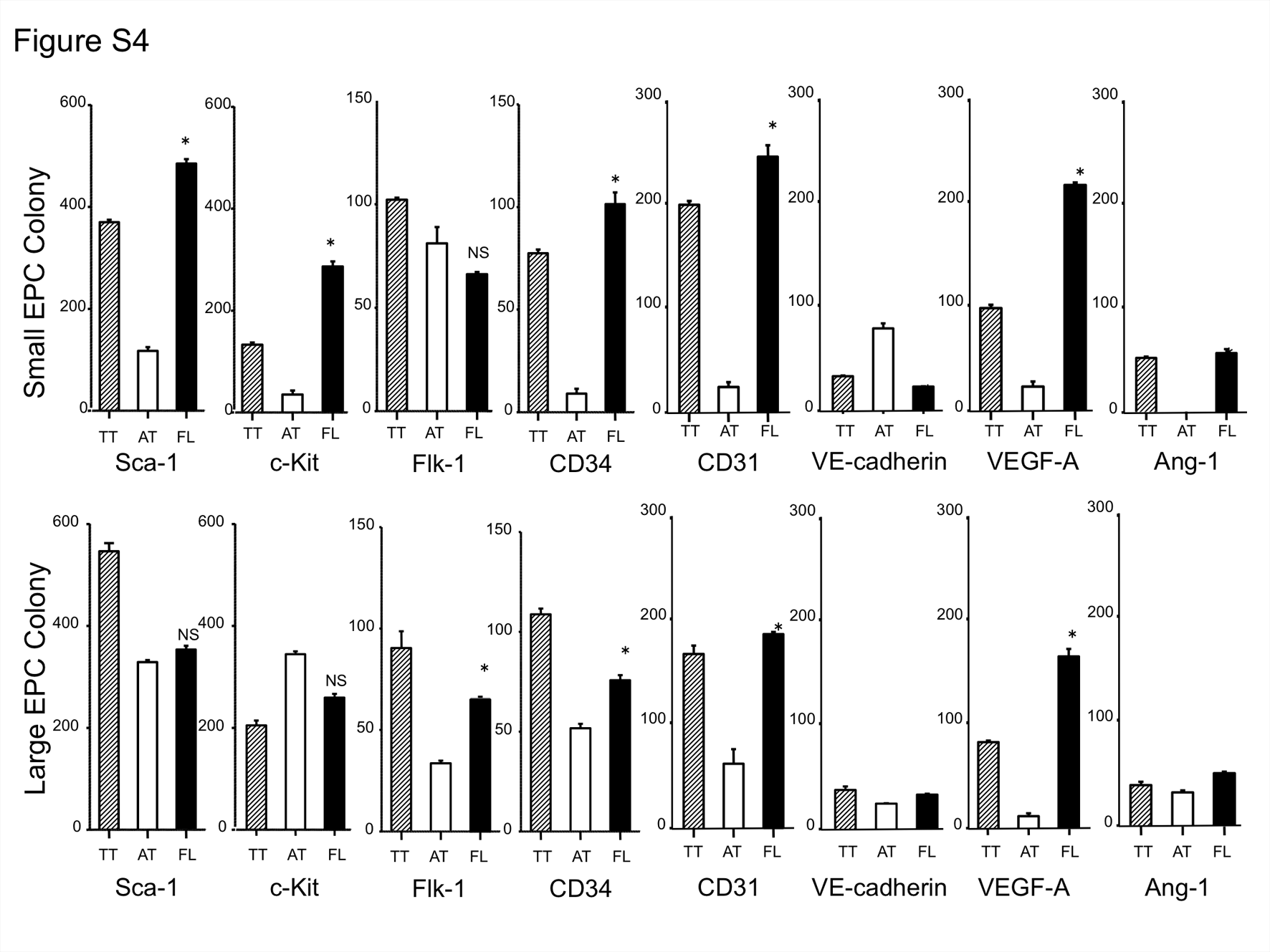

Supplement: Figure S4 — FL cell-derived EPC colonies exhibit EPC-like characteristics. FL cell-derived small EPC colony expressed higher mRNA of stem/progenitors and FL cell-derived large EPC colony expressed higher mRNA of endothelial cell and cytokines than AT cell-derived colonies. TT cells, AT cells and FL cells are seeded in the methylcellulose-based medium and cultured. After 14 days in culture, small EPC colonies (Small) and large EPC colonies (Large) were manually aspirated by glass capillary for real-time RT-PCR analysis. All mRNA expressions were normalized to GAPDH and presented in the graphs. Y-axis: relative mRNA expression to GAPDH. * and NS, P<0.05 vs. AT. (TIFF) [file pone.0028639.s004.tif]

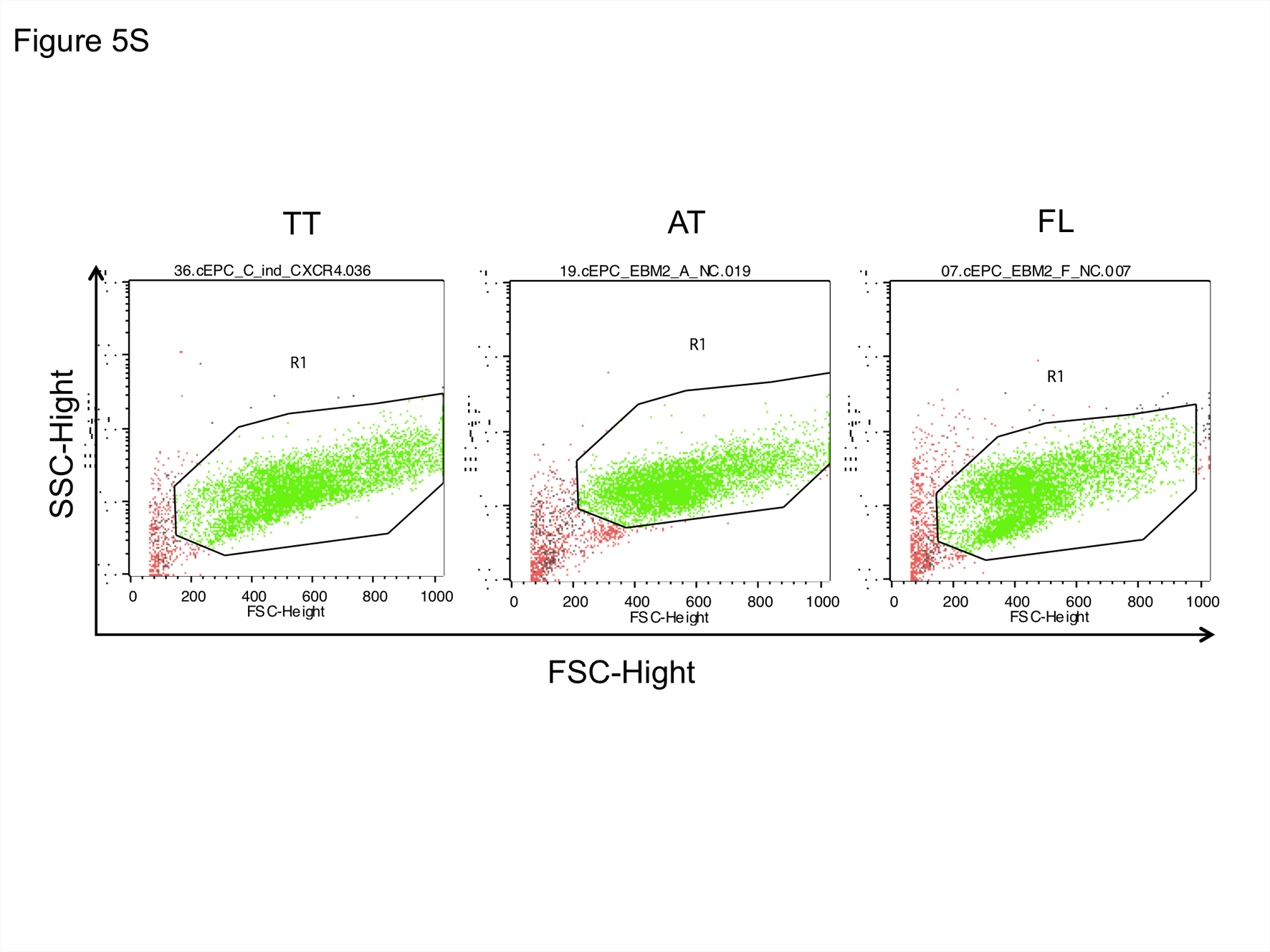

Supplement: Figure S5 — Forward Scatter (FSC) and Side Scatter (SSC) in TT, AT and FL for FACS analysis. After bone marrow derived mononuclear cells culture, the cells were harvested and examined the cell size by FSC and architecture by SSC to determine a certain cell population that is supposed to be analyzed for cell surface markers by FACS system. The gated cell population (green dots in R1 area) in each representative FSC/SCC graph was used for FACS analysis. (TIFF) [file pone.0028639.s005.tif]

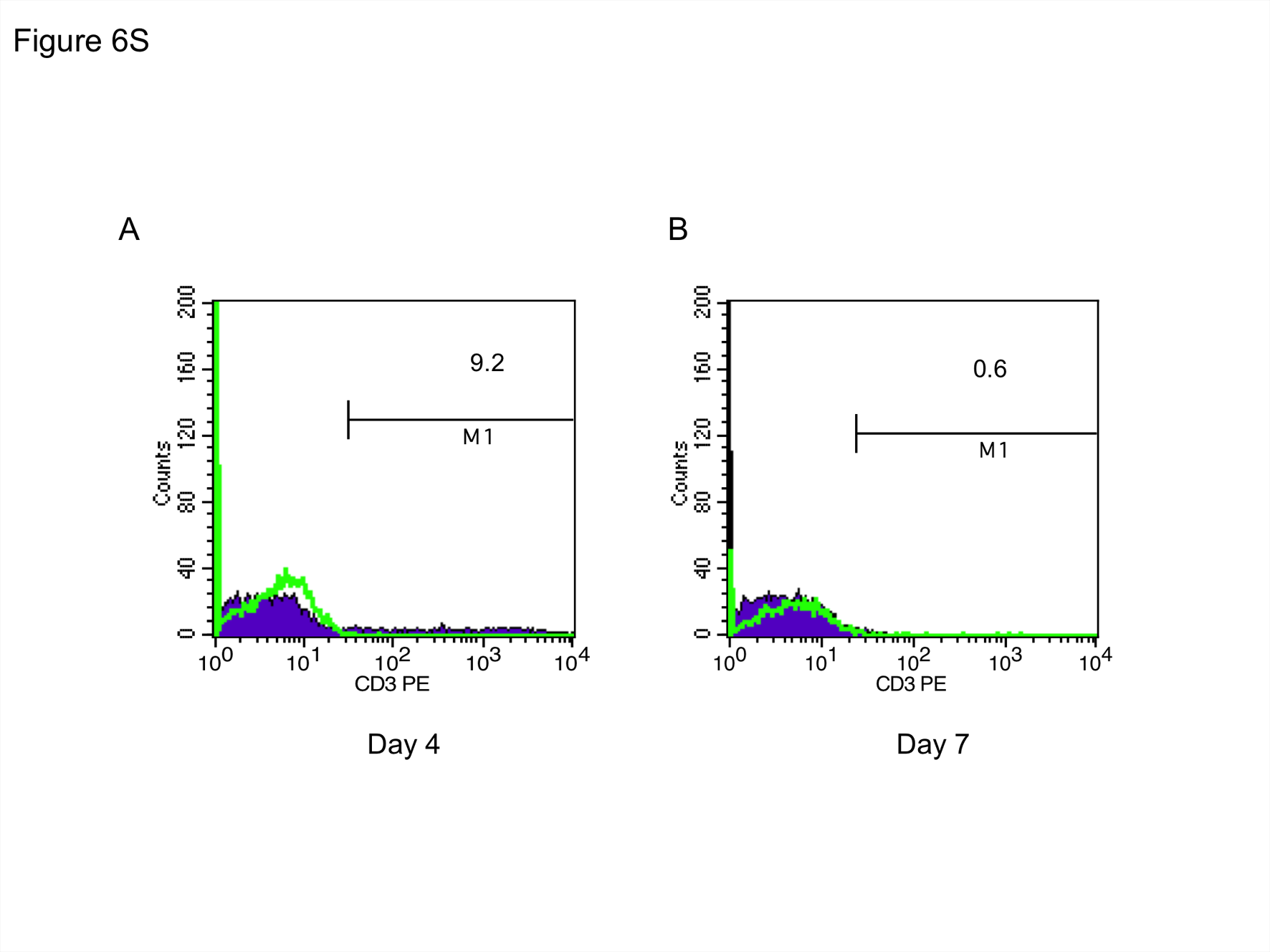

Supplement: Figure S6 — CD3 expression in TT cells at day4 and day7 after culture. After 4 days and 7 days in culture, adherent bone marrow derived mononuclear cells (TT cells) were harvested and analyzed for CD3 expression by FACS analysis. The percentage of CD3 positive cells in day 4-TT cells (A) and day 7-TT cells (B) was indicated in each graph. Purple area, Isotype control PE conjugated IgG and Green line, PE conjugated anti-CD3 antibody. (TIFF) [file pone.0028639.s006.tif]

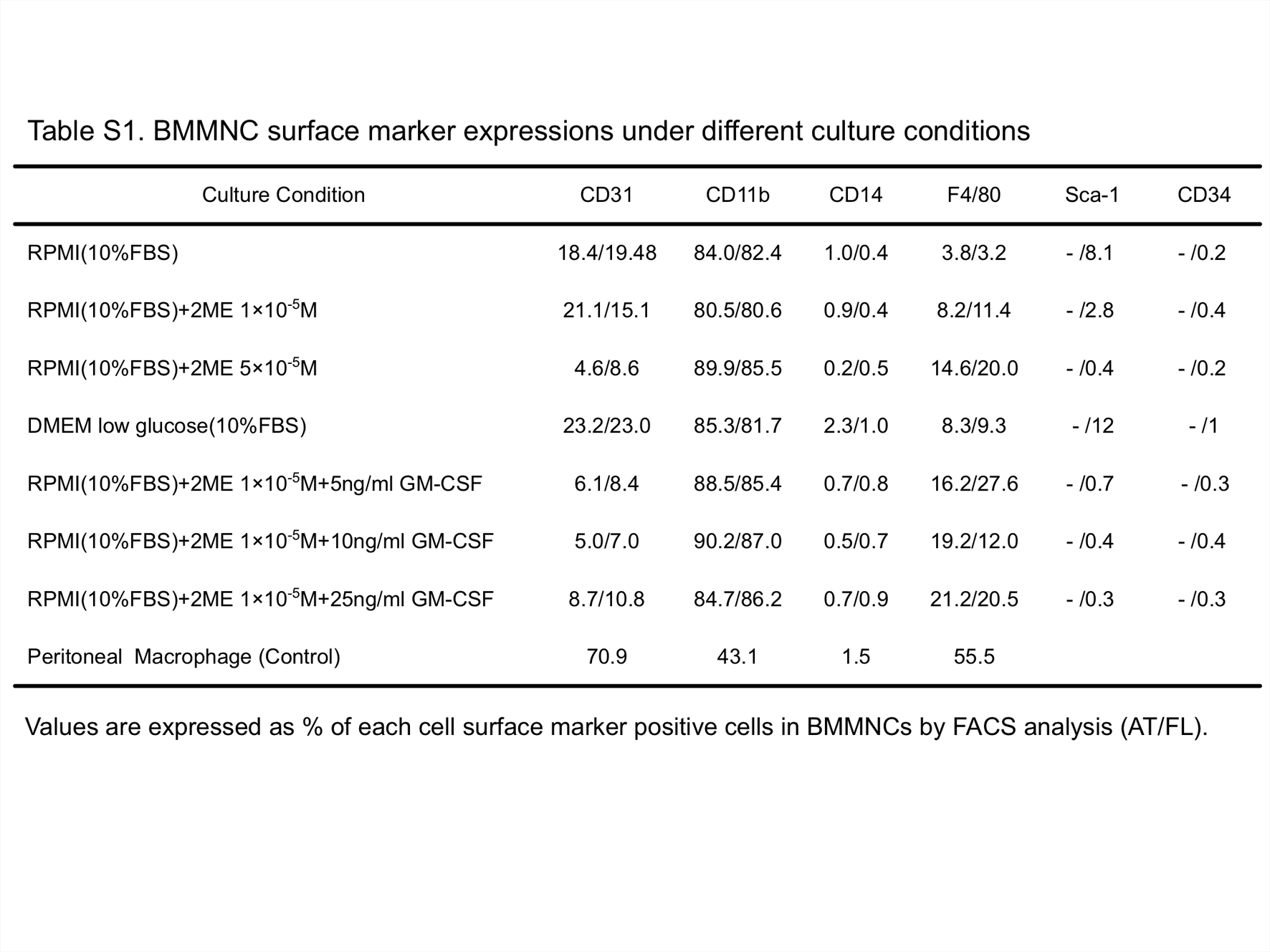

Supplement: Table S1 — BMMNC surface marker expressions under different culture conditions. (TIFF) [file pone.0028639.s007.tif]
